# Supplementary material for: Cloning and Functional Verification of CYP408A3 and CYP6CS3 Related to Chlorpyrifos Resistance in the Sogatella furcifera (Horváth) (Hemiptera: Delphacidae)
Source: Biology (Basel). 2021 Aug 18;10(8):795. doi: 10.3390/biology10080795 (PMC8389683; doi:10.3390/biology10080795)
Supplement: Supplementary file 1 [file biology-10-00795-s001.zip › biology-1334558-supplementary.pdf]

Supplementary Table S1. The primers of insecticide resistant-related P450 genes.

Types of primers	Primer	Sequence (5'-3')	Length
RT-qPCR	*CYP408A3-F*	CGGCAGCTCCCATACTGATT	105
	CYP408A3-R	GCAGACCGTAGATAGGCACC	
	CYP6CS3-F	TGTACCCTTGGTCCTGTGGA	159
	CYP6CS3-R	CAGGGATGTCAGTGGGTGTC	
	CYP4CE3-F	CCTGTGTACGGTTGTGCTGA	135
	CYP4CE3-R	AAAGTCGGCGAGTGTCCAAA	
	CYP4DD1-F	CGGATGCTCGGCAGTTCTAA	145
	CYP4DD1-R	TCCAGCACTGAATGGACAGT	
	CYP6AX3-F	CAGCGCTACTGATCCGATGT	150
	CYP6AX3-R	GCATTCTCCACTTCTCGCCT	
	CYP6AY3-F	GACGAGGGAATTGAGGCGAA	132
	CYP6AY3-R	TCTTGCCAACTCGTACAGGC	
	CYP6ER4-F	CGGCAGCTCCCATACTGATT	105
	CYP6ER4-R	GCAGACCGTAGATAGGCACC	
	CYP6FJ3-F	AGCGTTCTGGGAGTCCTGAT	148
	CYP6FJ3-R	TGAAGCTCTCTTTGGAGCGT	
	CYP417A4-F	GGCCTCCAACAGTTCCCATT	123
	CYP417A4-R	CCAGCCAAACTCTGACGGTA	
	CYP418A2-F	TGTACCCTTGGTCCTGTGGA	159
	CYP418A2-R	CAGGGATGTCAGTGGGTGTC	
Full-length primer	*CYP408A3-F*	TCATAAGATTCCACATATACAAT	1491
	CYP408A3-R	AAATTCCACTGGAAATTGAGAA	
	CYP6CS3-F	ATGGAACTTAAGAAGTACATCAA	1460
	CYP6CS3-R	TTACAGCTTTTCGAACCTAGCAT	
Reference gene	*RPL9F*	TGTGTGACCACCGAGAACAACTCA	142
	RPL9R	ACGATGAGCTCGTCCTTCTGCTTT	

Supplementary Table S2. This study used in vitro primers for dsRNA synthesis of insecticide resistant-related P450 genes.

RNAi	*dsCYP6CS3-F*	TAATACGACTCACTATAGGGACTTGTCCTCAGTTTGGCAAT	715
	dsCYP6CS3-R	TAATACGACTCACTATAGGGCCGAAAACATGCGGTAACCC	
	dsCYP408A3-F	TAATACGACTCACTATAGGGTGGTGGTGATTCTGTTACTGT	708
	dsCYP408A3-R	TAATACGACTCACTATAGGGACAGTCGGACTTATGAGCGT	

Supplementary Table S3. The dsRNA synthesis concentration of P450 gene

**Gene**	**Concentration(ng/μL)**
dsCYP6CS3	1193.7
	1865.4
	2021.1
dsCYP408A3	2508.5
	2337.0
	2465.7
